# Novel method to detect microRNAs using chip-based QuantStudio 3D digital PCR

**DOI:** 10.1186/s12864-015-2097-9

**Published:** 2015-10-23

**Authors:** Davide Conte, Carla Verri, Cristina Borzi, Paola Suatoni, Ugo Pastorino, Gabriella Sozzi, Orazio Fortunato

**Affiliations:** Tumor Genomics Unit, Department of Experimental Oncology and Molecular Medicine, Fondazione IRCCS Istituto Nazionale dei Tumori, via Venezian 1, 20133 Milan, Italy; Thoracic Surgery Unit, Fondazione IRCCS Istituto Nazionale dei Tumori, via Venezian 1, 20133 Milan, Italy

**Keywords:** miRNA, Absolute quantification, Lung cancer

## Abstract

**Background:**

Research efforts for the management of cancer, in particular for lung cancer, are directed to identify new strategies for its early detection. MicroRNAs (miRNAs) are a new promising class of circulating biomarkers for cancer detection, but lack of consensus on data normalization methods has affected the diagnostic potential of circulating miRNAs. There is a growing interest in techniques that allow an absolute quantification of miRNAs which could be useful for early diagnosis. Recently, digital PCR, mainly based on droplets generation, emerged as an affordable technology for precise and absolute quantification of nucleic acids.

**Results:**

In this work, we described a new interesting approach for profiling circulating miRNAs in plasma samples using a chip-based platform, the QuantStudio 3D digital PCR. The proposed method was validated using synthethic oligonucleotide at serial dilutions in plasma samples of lung cancer patients and in lung tissues and cell lines.

**Conclusion:**

Given its reproducibility and reliability, our approach could be potentially applied for the identification and quantification of miRNAs in other biological samples such as circulating exosomes or protein complexes. As chip-digital PCR becomes more established, it would be a robust tool for quantitative assessment of miRNA copy number for diagnosis of lung cancer and other diseases.

**Electronic supplementary material:**

The online version of this article (doi:10.1186/s12864-015-2097-9) contains supplementary material, which is available to authorized users.

## Background

MicroRNAs (miRNAs) are small non-coding RNAs, 19–24 nt-long, tissue specific, that regulate gene expression by post-trascriptional regulation [1, 2]. They play a critical role in development and differentiation processes of tissues and organs and are aberrantly expressed in different kinds of cancer [3, 4]. Over the last few years several studies have shown that miRNAs can be detected within body fluids such as plasma [5], serum [6], sputum [7], saliva [8], and urine [9]. Although the mechanism of secretion and incorporation of miRNAs has not been fully clarified, circulating miRNAs may play a pivotal and general role as signaling molecules in physiological and pathological events [10].

Notably, circulating miRNAs levels were found to correlate with cancer progression, therapeutic response, and patient survival, suggesting that they could also be used as non-invasive biomarkers [11, 12].

Lung cancer is the leading cause of cancer deaths in the world due to its high incidence and mortality, with 5-year survival estimates around 15 % for non-small-cell lung cancer (NSCLC) [13]. Despite recent advances in the management of lung cancer and the use of molecular targeted agents in specific clinical settings, the cure rate remains low due to drug-refractory recurrent disease [13].

We previously identified in tumor, normal lung tissue and plasma samples miRNA signatures with diagnostic and prognostic potential [14]. In a recent study we showed that the combination of low dose computed tomography (LDCT) screening and our plasma miRNA signatures reduced LDCT false positives in a retrospective screening series of more than 1000 individuals [15].

The high potential of the circulating miRNAs as molecular marker of disease is diminished by a lack of consensus regarding an optimal method of normalization in plasma samples. The small-nucleolar RNAs (snoRNAs), such as RNU6B and RNU48, cannot be used to this purpose due to the absence in plasma [16]; other housekeeping miRNAs candidate have been proposed in different studies but a global consensus on their use is still lacking [17, 18]. Alternatively, for assay with a relatively large number of miRNAs, a normalization using the mean or the median could be applied [19]. Our group described an approach for circulating miRNAs profiling in plasma samples based on the evaluation of 24 miRNAs reciprocal levels measured by quantitative Real-Time PCR [20]. Recently, it was suggested an absolute quantification of miRNAs using RT-PCR with a standard curve generated with a synthetic oligonucleotide but this approach could be useful only for individual miRNA quantification but not for multiplex miRNA evaluation. Furthermore, other groups proposed a normalization method based on the addition of a spike-in miRNAs. They recommended the use of *C. elegans* control miRNAs during the denaturation of samples to normalize the variability that could affect the reaction efficiency [21].

Digital PCR (dPCR) is an end-point PCR method that is used for absolute quantification. The dPCR concept was conceived in 1992 [22] and it was used to quantify KRAS mutations in DNA from colorectal cancer patients [23]. Digital PCR has many potential applications, including the detection and quantification of low-level pathogens [24], rare genetic sequences [25], copy number variations (CNVs) [26], gene expression in single cells [27] and quantification of circulating miRNAs expression [28, 29].

**Fig. 1:**
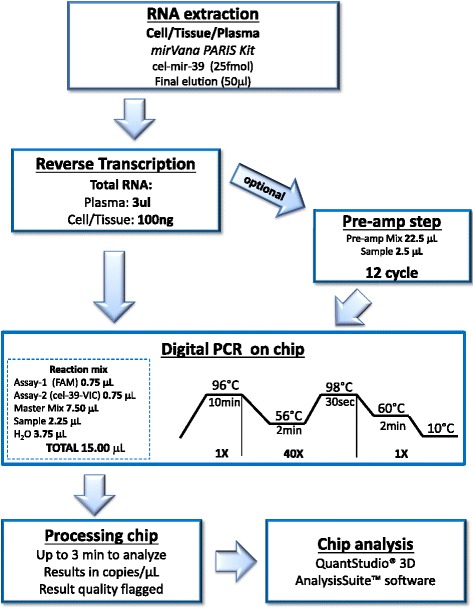
Workflow of a digital PCR experiment

In this work, we propose for the first time, a methodological workflow, shown in Fig. 1, for the absolute quantification of miRNAs, in plasma or tissue/cells of lung cancer patients using a chip-based platform, the QuantStudio 3D Digital PCR. For the description of the results we adopted the guidelines for the publication of digital PCR experiments described by Huggett et al. [30].

This innovative data analysis tool allowed us to circumvent the normalization issue and given the high reproducibility of this procedure, we believe that it could be routinely used for the analysis of miRNAs also in cancer clinical series.

## Methods

### Samples

#### Plasma samples

Plasma samples were collected from high-risk heavy smoker volunteers aged from 50 to 75 years, including current or former smokers with a minimum pack/year index of 30 enrolled in a LDCT screening trial (BioMild) performed at our Institution [31].

#### Lung cancer cell lines

Human lung cancer cell lines, A549 and H1299, were obtained from the American Type Culture Collection (ATCC). LT73 cells were derived in our laboratory from a primary lung tumor of a 68–year old Caucasian male with lung adenocarcinoma [32].

#### PDX

Lung cancer patient’s derived xenografts (PDXs) were developed by directly implanting fragments of the patient’s living tumor in the flanks of immunocompromised mice [33].

#### Ethics statement

Tissue and plasma specimens were obtained according to the Internal Review and the Ethics Boards of the Istituto Nazionale Tumori of Milan (INT 2111). All patients provided informed consent.

### MicroRNA profiling

#### Taqman assays

MiRNA expression was analyzed using Taqman MicroRNA assays (Thermo Fisher Scientific) : mir-16-5p (ID:000391), mir-21-5p (ID:000397), mir-126-3p (ID:002228), mir-486-5p (ID:001278), mir-660-5p (ID:001515), cel-mir-39 (CUSTOM) and RNU48 (ID:001006).

#### Plasma

Total RNA was extracted from 200 μl plasma samples using mirVana PARIS Kit (Thermo Fisher Scientific), according to the protocol for biological fluids. Synthetic *C. elegans* miRNA-39 (cel-miR-39) was used as spiked-in control, adding to each plasma sample 5 μl from a 5 fmol/ μl stock tube (Qiagen). Samples were eluted in 50 μL of Elution Solution pre-heated at 95 °C to obtain more concentrated total RNA.

RT reaction was performed on 3 μl of total RNA, using the TaqMan MicroRNA Reverse Transcription Kit and a Custom TaqMan RT Primer Pool (Thermo Fisher Scientific), according to the manufacturer’s instruction. Since we started from a small amount of total RNA, a pre-amplification step of 12 cycle was required, thus 2.5 μL of each RT product were pre-amplified using a Custom TaqMan PreAmp primer pool (Thermo Fisher Scientific).

#### Tissues and cultured cells

PDX tissue samples were disrupted and homogenized using 3 mm Tungsten Carbide Beads and the Mixer Mill MM300 (Qiagen). For tissue samples and cultured cells, total RNA was extracted using mirVana PARIS Kit (Thermo Fisher Scientific) following manufacturer’s instructions. Cel-miR-39 was used as spiked-in control, by adding to each samples 5 μl from a 5 fmol/ μl stock tube. Total RNA was quantified with the NanoDrop 2000 (Thermo Fisher Scientific).

Starting from 100 ng of total RNA, reverse transcription was performed using the TaqMan microRNA Reverse Transcription Kit and a TaqMan RT Primer Pool with the miRNAs of interest according to the manufacturer’s instruction (Thermo Fisher Scientific).

#### PCR on chip

We combined 2,25 μl of RT product (RT or PreAmp product obtained in the previous step) with 3,75 μl nuclease-free H_2_O, 7,50 μl QuantStudio™ 3D Digital PCR Master Mix, 0,75 μl of TaqMan MicroRNA Assay-1 (20X) and 0,75 μl TaqMan MicroRNA Assay-2 20X (cel-mir-39-VIC).

To avoid pipetting errors, we prepared a stock solution and we included 10 % excess for volume loss from pipetting. This sample mix was added on each chip and loaded on ProFlex™ 2x Flat PCR System with the following program (Table 1):

**Table 1:** PCR run protocol

Step type	Time	Temperature (°C)
Hold	10 min	96
Cycle (40 cycles)	2 min	56
	30 s	98
Hold	2 min	60
Hold	∞	10

Absolute quantification was determined using QuantStudio 3D Digital PCR System (Thermo Fisher Scientific) and analyzed with QuantStudio 3D AnalysisSuite Cloud Software (Thermo Fisher Scientific).

## Results and discussion

### Chip quality control

The software assesses whether the data on a chip is reliable based upon loading, signal, and noise characteristics and displays quality indicators for each chip in a project.

This quality control is based on the number of partitions that exceed the selected quality threshold (fixed automatically at 0.5) on the total number of wells filled correctly. To get a precise quantification we settled a threshold of 10.000 data points for quality control of the chip (Additional file 1: Figure S1).

### Use of spike-in (VIC fluorescence)

Several studies report on the use of synthetic RNA or miRNA molecules as spike-in controls for mRNA/miRNA expression data normalization [34–36].

In the proposed method, we decided to add in the mix an exogenous spike-in miRNA (cel-mir-39 coniugated with VIC fluorescence) as internal control for efficiency of the whole reaction (from extraction to PCR) since the fluorescence analysis using only FAM probes did not allow a precise quantification of miRNAs.

**Fig. 2:**
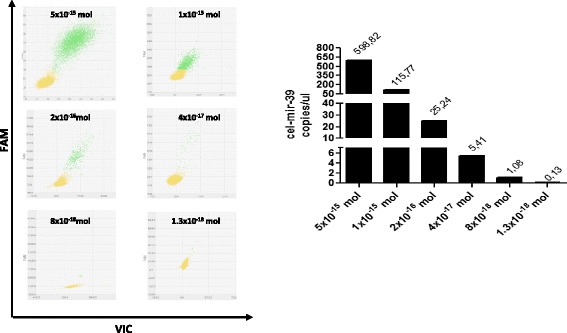
Quantification of synthetic oligonucleotides by dPCR. Analysis of five-fold dilution series of cel-mir-39 using FAM and VIC probes. Representative plot of dPCR (left panels) and number of copies/ul (right panel) were shown. The data points in the plot are color-coded according to the following call types: FAM (blue), VIC (red), FAM + VIC (green) and NOT AMPLIFIED (yellow)

To test the overall performance of the method in terms of efficiency, precision, and sensitivity we generated a standard curve using a serial dilution of cel-mir-39 mimic (Qiagen). We generated a curve consisting in six points of a five-fold serial dilution, starting from 5 fmol of cel-mir-39 mimic. In this way the copy number of each single miRNAs measured displayed a good linear response to input mimic amount (Fig. 2). Furthermore, we calculated the expected copies for each dilution of cel-mir-39 as described *by Hindson* et al. [37] to show dPCR detection efficiency assuming 100 % RT efficiency and an accurate known concentration of the synthetic miRNA supplied by the vendor (Table 2). We noted that absolute measurements by dPCR corresponded to 74–115 % of the theoretically input copies, indicating that absolute detection by dPCR is remarkably efficient.

**Table 2:** dPCR detection efficiency

cel-mir-39 Dilution	miRNA copies/ul expected	miRNA copies/ul observed	Detection efficiency (%)
D1	500	598	83
D2	100	115	87
D3	20	25,24	78
D4	4	5,4	74
D5	0,8	1,08	74
D6	0,15	0,13	115

### miRNA expression analysis

**Fig. 3:**
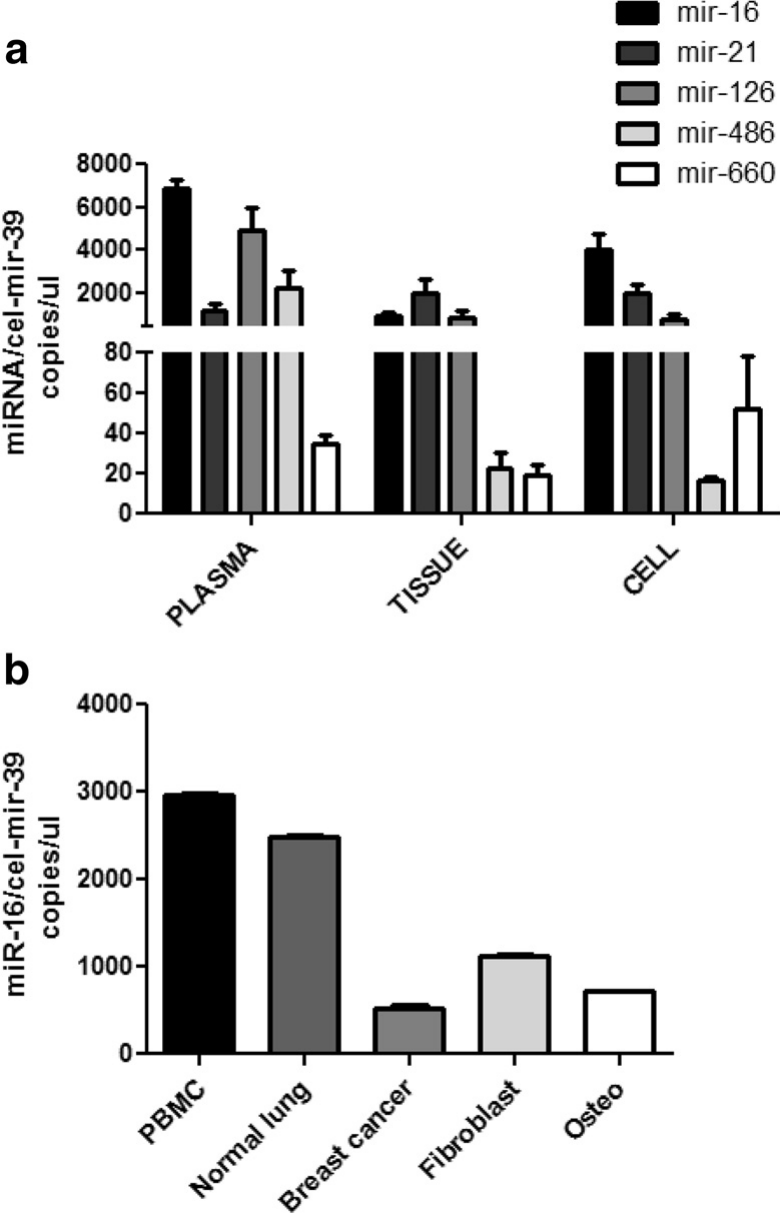
Absolute quantification of miRNA expression levels. **a** Bar graphs show the number of copies/ul for mir-16-5p, mir-21-5p, mir-126-3p, mir-486-5p and mir-660-5p in three different samples (plasma, tissue and cells) (*n* = 3). Data are expressed mean ± standard error of the mean. **b** mir-16-5p expression in different specimens: healthy peripheral blood mononuclear cells (PBMC), normal lung tissues and cell lines from breast cancer, fibroblast and osteosarcoma. The experiments were done in duplicates

We decided to use our methodology for the quantification of miRNAs levels in different type of samples (plasma, tissue and cells) and its potential utility for cancer diagnosis of our 24 miRNAs composing the diagnostic signature [15]. As shown in Fig. 3a, we analyzed the expression of five miRNAs (mir-16-5p, mir-21-5p, mir-126-3p, mir-486-5p and mir-660-5p) already demonstrated to have a role in lung cancer development [32, 38–41]. This proposed method could be useful for the quantification of miRNA levels in various samples from normal or pathological conditions. To demonstrate the potential use of this protocol, we analyzed the expression of mir-16-5p, an ubiquitous miRNA, in different specimens as healthy peripheral blood mononuclear cells (PBMC), normal lung tissue and cell lines of breast cancer, fibroblast and osteosarcoma (Fig. 3b).

### Plasma analysis

**Fig. 4:**
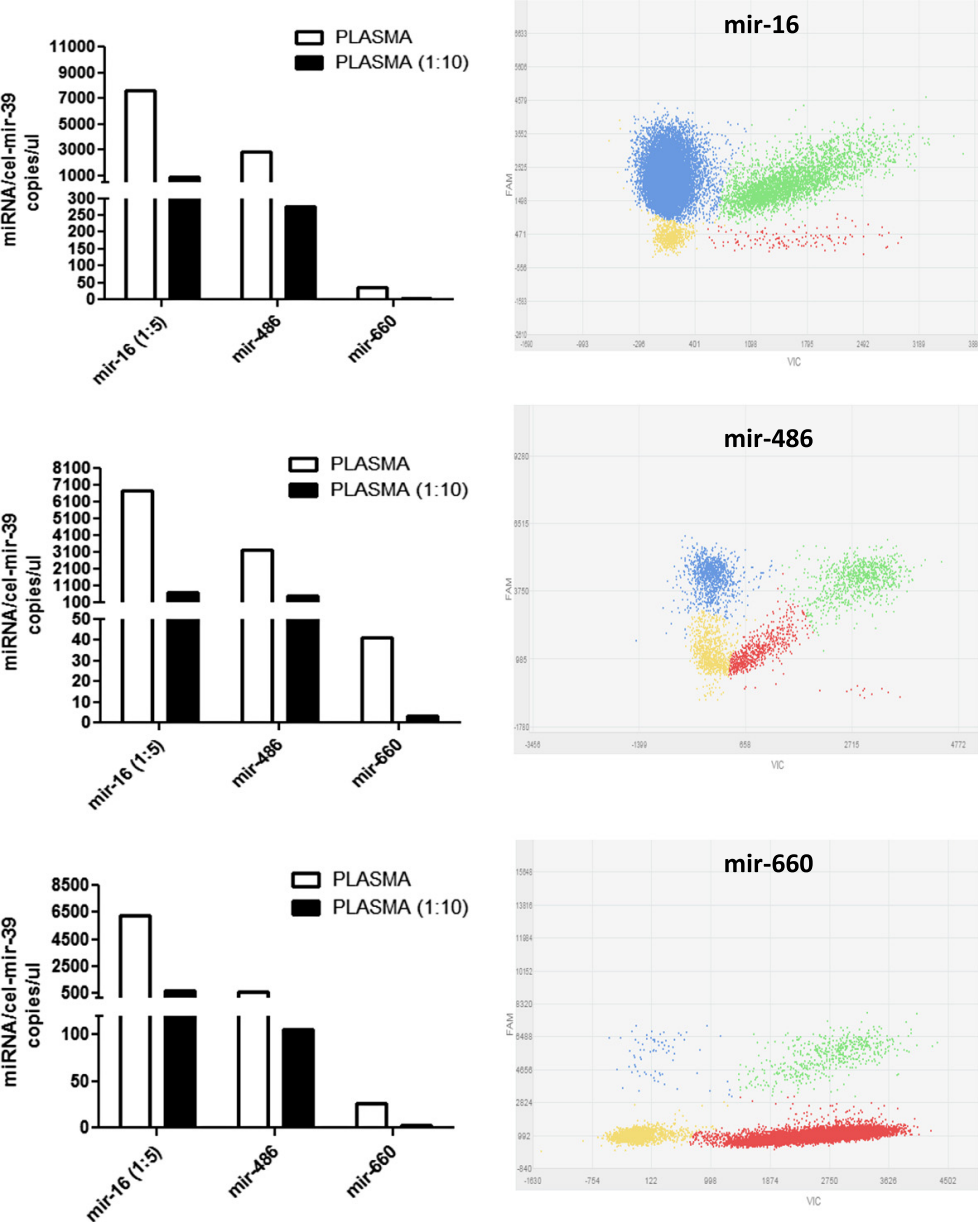
miRNAs expression levels in plasma samples. Bar graphs (left) show number of copies/μl of each miRNAs analyzed of three different plasma samples and their respective ten-fold dilution. Representative dPCR plots of the miRNA analysis (right). The data points in the plot are color-coded according to the following call types: FAM (blue), VIC (red), FAM + VIC (green) and NOT AMPLIFIED (yellow)

To evaluate the sensitivity and the precision of our digital PCR methods, we select three of the selected miRNAs showing different levels of expression (i.e. mir-16-5p mir-486-5p and mir-660-5p) and analyzed their expression in plasma samples. On the basis of the expected circulating miR-16-5p levels, we performed a five-fold dilution of the samples to have high efficiency of quantification compared to the undiluted sample (data not shown for undiluted sample). For the other two miRNAs, mir-486-5p and mir-660-5p, no dilution was required (Fig. 4 and Table 3). To compare results obtained with qPCR, we tried to correlate raw Ct data for these five circulating miRNA obtained with custom microfluidic cards [20] and the number of copies obtained with dPCR (Table 4) assuming 100 % primers efficiency as described by the vendor. As reported in Table 4, we observed a good correlation intra-run assay and between samples for circulating miRNAs analyzed.

**Table 3:** Absolute quantification of miRNA levels in plasma

Sample	Assay	cel-mir-39	CI- cel-mir-39	Target miRNA	CI- miRNA	Data points above threshold (0.5)
PL 0 (1:5)	16–39	237,6	229,8 -- 245,6	3748,5	3667,2 -- 3831,7	18,686 of 19,678
PL 0	486-39	3438,1	3364,5 -- 3513,4	4120,8	4020,8 -- 4223,3	17,202 of 19,331
PL 0	660-39	2588,9	2539,8 -- 2638,9	40,32	37,223 -- 43,672	17,564 of 19,417
PL 0 (1:5) 1:10	16–39	24,3	21,8 -- 27,0	387,8	376,6 -- 399,4	15,745 of 16,444
PL0 1:10	486-39	184,9	177,6 -- 192,5	175,2	168,2 -- 182,6	16,184 of 17,874
PL 0 1:10	660-39	567,6	553,93 -- 581,54	8,56	7,2– 10,2	17,082 of 19,260
PL 1 (1:5)	16–39	392,2	381,6 -- 403,2	7856	7365,6 -- 8379	17,875 of 19,224
PL1	486-39	2266,7	2224,4 -- 2309,7	4295,9	4187,9 -- 4406,6	17,220 of 18,865
PL 1	660-39	839,7	819,5 -- 860,4	20,42	17,9 -- 23,2	13,137 of 16,404
PL 1 (1:5) 1:10	16–39	46,1	42,8 -- 49,8	1043,7	1022,8 -- 1065	16,922 of 17,932
PL1 1:10	486-39	125,7	119,9 -- 131,8	394,12	383,1 -- 405,4	16,758 of 17,839
PL1 1:10	660-39	115,8	110,4 -- 121,4	2,53	1,9 -- 3,5	17,818 of 19,142
PL 2 (1:5)	16–39	286,8	276,8 -- 297,2	6907,2	6535,1 -- 7300,5	13,768 of 15,156
PL2	486-39	1141,1	1114,9 -- 1168	567,2	551,1 -- 583,7	12,279 of 19,292
PL2	660-39	1305,0	1279,7 -- 1330,8	26,78	24,2 -- 29,6	16,422 of 19,687
PL 2 (1:5) 1:10	16–39	43,7	40,4 -- 47,2	877,4	859 -- 896,2	16,864 of 19,038
PL2 1:10	486-39	188,3	181,0 -- 195,9	112,8	107,3 -- 118,6	16,513 of 19,004
PL2 1.10	660-39	123,1	117,4 -- 129,0	1,9	1,4 -- 2,8	17,112 of 18,704

**Table 4:** Pearson correlation in plasma samples between assays and samples

Real Time PCR (Ct)	miR-16	mir-126	miR-486	mir-21	miR-660	Pearson correlation
PLASMA 1	17,86	19,87	20,83	24,63	28,57	−92 %
PLASMA 2	18,39	19,58	20,38	24,32	28,12	−89 %
PLASMA 3	18,59	18,88	21,35	24,66	28,76	−83 %
dPCR (copies/ul)	miR-16	mir-126	miR-486	mir-21	miR-660	
PLASMA 1	7590	4746,84	2882	755,79	37	
PLASMA 2	6766	3261,78	3201	1855,87	41	
PLASMA 3	6230	6840,79	643	913,46	27	
Pearson correlation	−99 %	−76 %	−93 %	−98 %	−87 %	

To demonstrate the specificity of the absolute quantification, we performed a ten-fold dilution for each samples and we confirmed that there was a linearity for each miRNAs between the two samples (fold-dilution average between samples: mir-16-5p: 8,81 ± 0,675, mir-486-5p: 7,67 ± 2,41, mir-660-5p: 9,96 ± 1,76).

The number of copies per μl between samples was normalized based on the mean of cel-mir-39 expression for each chip and multiplied by the number of dilution folds in the plasma.

We replicated miRNAs expression analysis on three different plasma samples and we obtained similar data of expression for each miRNAs (Fig. 4 and Table 3). Negative and non-template controls for each miRNA were run on chip and did not show any positive results.

### Cell analysis

**Fig. 5:**
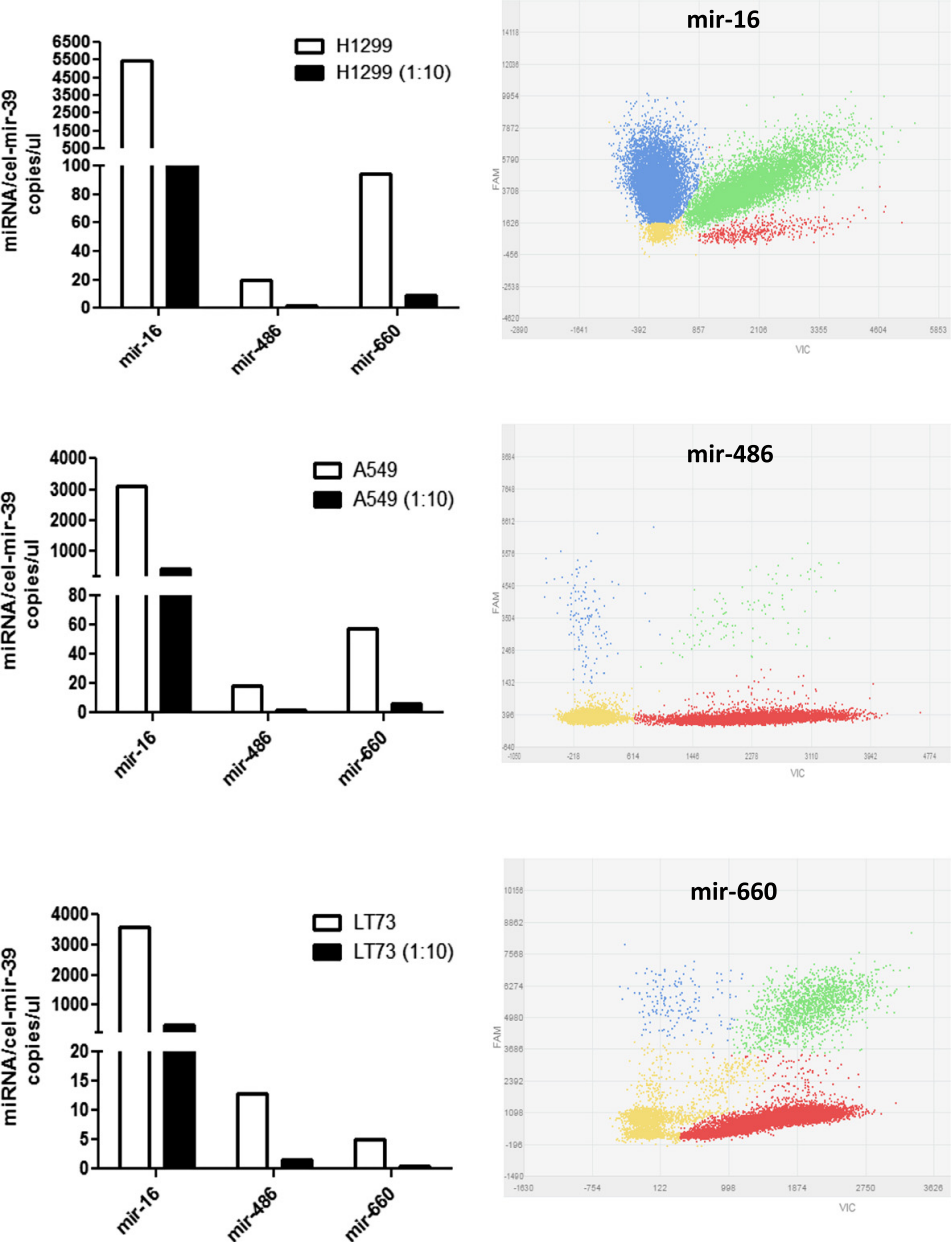
Absolute quantification of miRNAs in lung cancer cells. Bar graphs (left) show number of copies/μl of mir-16, mir-486 and mir-660 in H1299 (upper), A549 (middle) and LT73 (lower) cell lines and their respective ten-fold dilution. Representative dPCR plots of the miRNA analysis (right). The data points in the plot are color-coded according to the following call types: FAM (blue), VIC (red), FAM + VIC (green) and NOT AMPLIFIED (yellow)

To demonstrate the potential use of QuantStudio 3D digital PCR for cellular miRNAs expression analysis, we analyzed the levels of these miRNAs in three different lung cancer lines. As already described, mir-16-5p is one of the most abundant miRNA in lung cancer cells, whereas mir-660 and mir-486-5p expression is very low. Starting from 100 ng of total RNA, we quantified the miRNA expression levels using undiluted or ten-fold diluted samples (Fig. 5 and Table 5). As shown in the graphs the sensitivity of the method was confirmed also for miRNA cellular expression (fold-dilution average between samples: mir-16-5p: 10,45 ± 3,87, mir-486-5p: 12,45 ± 2,95, mir-660-5p: 10,99 ± 3,69). Moreover, we performed RNU48 analysis cell samples and we observed that the number of copies for RNU48 was similar for all the samples starting from the same amount of input RNA (data not shown). Negative and non-template controls for each miRNA were run on chip and did not show any positive results

**Table 5:** Number of miRNAs copies in lung cancer cell lines

Sample	Assay	cel-mir-39	CI- cel-mir-39	Target miRNA	CI- miRNA	Data points above threshold (0.5)
H1299	16–39	981,8	962,5 -- 1001,5	5025,6	4880,6 -- 5175	18,079 of 18,671
H1299	486-39	940,9	921,7 -- 960,6	17,1	15,1 -- 19,4	17,084 of 19,738
H1299	660-39	1.275,7	1252 -- 1299,8	112,7	107,5 -- 118,2	18,148 of 19,385
H1299 1:10	16–39	302,9	293,4 -- 312,8	439,3	427,4 -- 451,5	16,281 of 18,954
H1299 1:10	486-39	345,8	335,8 – 356,1	1,8	1,2 -- 2,6	17,328 of 18,723
H1299 1:10	660-39	334,3	324,4 -- 344,5	9,2	7,7 -- 10,9	16,976 of 18,096
A549	16–39	808,9	790,1 -- 828,3	2130,9	2087,6 -- 2175,1	14,270 of 19,057
A549	486-39	1309,6	1285,3 -- 1334,3	19,9	17,8 -- 22,3	18,008 of 19,310
A549	660-39	1403,2	1372,5 -- 1434,6	69	64,0 -- 74,0	12,610 of 17,988
A549 1:10	16–39	486,6	473,6 -- 499,9	483,4	470,5 -- 496,7	15,486 of 16,723
A549 1:10	486-39	401,3	379,9 -- 423,8	1,57	0,7 -- 3,5	17,285 of 18,655
A549 1:10	660-39	367,4	355,9 -- 379,5	5,36	4,1 -- 6,8	13,841 of 17,322
LT73	16–39	655,2	640,7 -- 670,1	3266,7	3200,2 -- 3334,5	18,070 of 19,251
LT73	486-39	921,1	902,2 -- 940,4	16,5	14,6 -- 18,8	17,170 of 18,120
LT73	660-39	574,0	558,7 -- 589,8	4,0	3,0 -- 5,3	13,606 of 19,582
LT73 1:10	16–39	90,4	85,5 -- 95,5	409,9	398,6 -- 421,5	16,673 of 18,990
LT73 1:10	486-39	66,8	62,7 -- 71,1	1,3	0,9 -- 2,1	17,359 of 18,326
LT73 1:10	660-39	60,8	57,0 -- 64,8	0,3	0,1 -- 0,8	18,164 of 19,404

### PDX analysis

**Fig. 6:**
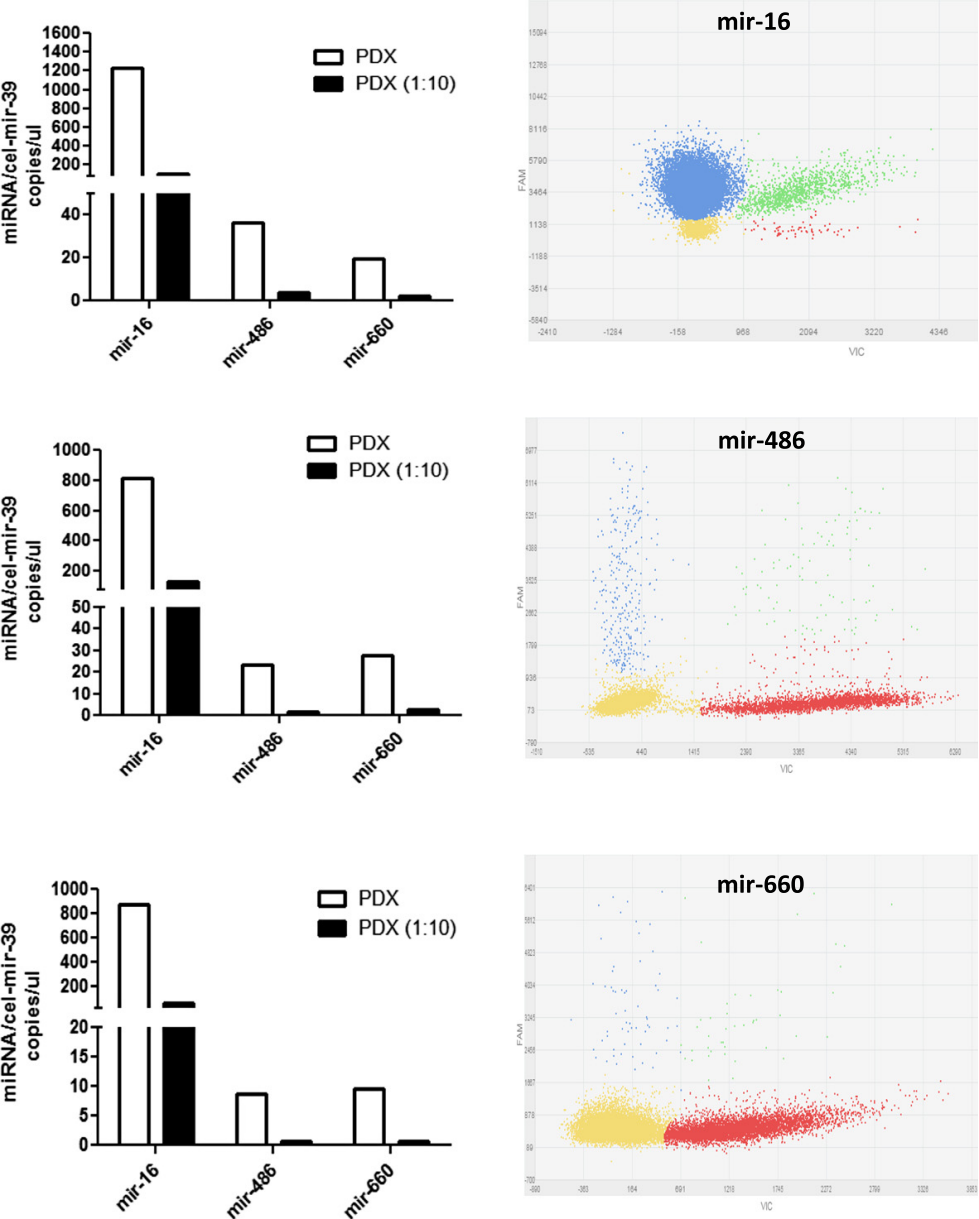
Number of miRNAs copies per μl in lung PDX tissues. Bar graphs (left) show the expression levels of the miRNAs analyzed in three different samples of PDX and their respective ten-fold dilution. Representative dPCR plots of the miRNA analysis (right). The data points in the plot are color-coded according to the following call types: FAM (blue), VIC (red), FAM + VIC (green) and NOT AMPLIFIED (yellow)

Our method could be applied for the analysis of miRNAs expression in tissues and to demonstrate this potential use we performed the same analysis described above. We extracted RNA and measured miRNAs levels from three different samples of our patients derived xenografts. As described in Fig. 6 and Table 6, mir-16 was the most expressed miRNAs in tissues whereas mir-486 and mir-660 had similar expression levels. Ten fold dilution of the samples confirmed the sensitivity of the analysis in tissues (fold-dilution average between samples: mir-16-5p: 9,92 ± 2,18, mir-486-5p: 10,39 ± 1,32, mir-660:-5p 11,03 ± 1,92). As described above, we performed RNU48 analysis on all tissues and we observed that the number of copies for RNU48 was similar for all the samples starting from the same amount of input RNA (data not shown). Negative and non-template controls for each miRNA were run on chip and did not show any positive results.

**Table 6:** miRNAs levels in PDXs tissues

Sample	Assay	cel-mir-39	CI- cel-mir-39	Target miRNA	CI- miRNA	Data points above threshold (0.5)
PDX 1	16–39	430,6	418 -- 443,5	968,4	946,9 -- 990,4	14,289 of 18,775
PDX1	486-39	682,8	666,8 -- 699,1	45,2	41,8 -- 48,9	15,843 of 18,068
PDX 1	660-39	521,2	507,9 -- 534,9	18,8	16,7 -- 21,2	16,106 of 18,346
PDX 1 1:10	16–39	122,4	105,8 -- 141,7	162,29	155,7 -- 169,2	18,068 of 19,395
PDX1 1:10	486-39	58,0	54,3 -- 62,1	2,93	2,2 -- 3,9	17,381 of 19,135
PDX1 1:10	660-39	52,7	47,5 -- 58,4	1,72	0,9 -- 3,0	17,868 of 18,826
PDX 2	16–39	601,9	586,6 -- 617,7	937,7	916,8 -- 958,9	14,472 of 16,749
PDX 2	486-39	532,9	520,1 -- 546,1	23,8	21,5 -- 26,4	17,831 of 19,264
PDX 2	660-39	441,6	426,6 -- 457,2	23,2	20,2 -- 26,5	10,233 of 15,163
PDX 2 1:10	16–39	59,7	55,9 -- 63,8	134,7	128,8 -- 140,9	17,341 of 19,135
PDX 2 1:10	486-39	57,5	53,2 -- 62,2	1,5	0,9 -- 2,4	13,040 of 18,311
PDX 2 1:10	660-39	62,3	58,3 -- 66,4	2,8	2,1 -- 3,8	17,358 of 19,226
PDX3	16–39	83,5	78,9 -- 88,4	798,1	781,0 -- 815,5	17,174 of 19,355
PDX3	486-39	104,7	98,6 -- 111,2	9,9	8,2 -- 12,0	12,183 of 18,035
PDX3	660-39	86,1	81,5 -- 91,1	9,0	7,6 -- 10,7	17,004 of 18,146
PDX3 1:10	16–39	12,9	11,2 -- 14,8	90,5	85,6 -- 95,5	17,089 of 18,817
PDX3 1:10	486-39	7,3	6,7-- 8,7	0,5	0,3 -- 1,0	17,966 of 19,844
PDX3 1:10	660-39	7,7	6,4 -- 9,2	0,5	0,3-- 1,1	17,509 of 19,282

### Precision of the methodology

To determine repeatability of the methods we performed mir-16-5p analysis on 5 different samples in duplicates and measured the coefficient of variation [37] in the same PCR run. The same samples were also analyzed in two different PCR runs to determine the overall precision of the methodology. As shown in Table 7, digital PCR results displayed low variation both intra and inter-run replicates (% CV within-run: 4 %; overall precision: 4 %) (Table 7).

**Table 7:** Coefficient of variation between dPCR replicates

	CV across intra-run replicates	CV across inter-run replicates
Sample 1	0,004	0,05
Sample 2	0,033	0,02
Sample 3	0,009	0,02
Sample 4	0,008	0,07
Sample 5	0,142	0,04
Average	0,039	0,040

## Conclusion

In this work, we developed for the first time a new method for the detection of miRNAs in different type of biological specimen as plasma, cell lysates or tissue. This method could be particularly useful for quantification of miRNAs in those biological samples, such as plasma/serum or exosomes, where lack of consensus for the normalization strategy prevents clinical applications.

Digital PCR has a great potential but several tips need to be followed: first a rough estimate of the concentration of your target of interest has to be previously done in order to make appropriate dilutions. Otherwise, it is possible that too many partition will get multiple copies preventing an accurate calculation of the copy number of your miRNA. Furthermore, non-template controls and a RT negative control must be set up for each miRNA when using a “primers pool methods” for retro-transcription.

A chip-based digital PCR approach has the advantage to require less pipetting steps and to reduce PCR contamination. In comparison, the droplet digital PCR requires multiple pipette transfers that could potentially increase the risk of contamination. Furthermore, QuantStudio 3D chips have 20.000 fixed reaction wells whereas droplet PCR rely upon the generation of droplets, a step that could be extremely variable. Overall, our proposed method for miRNA quantification using a chip-dPCR were comparable in terms of accuracy and precision to the study reported by Miotto et al. using droplet digital PCR. (% CV within-run: chip 4 % droplet 5.1; overall precision: chip 4 % and droplet 13 %) [42].

Obviously, this approach has some limitations, for example, the ability to perform only one sample per chip, although it is possible to load in the thermocycler up to 24 chips. Moreover, using this approach tests in multiplex fluorescence can be carried out but only with two probes per chip. To date, dPCR could be potential useful for clinical diagnostic purpose only for small scale samples but we believe that, during the next years, the improvement of the methodology could permit multiplexing analysis. At the time, the instrument is not able to perform an accurate analysis when there is only one fluorescence, VIC or FAM, because it is set to choose a threshold for both fluorescence. In our methods, to solve this technical problem, we decided to put a reference control, an exogenous spike-in with VIC probe, which allows an accurate miRNA copy number quantification and also to have control of the whole process. A new version of the analysis software, now released, permit manual modifications of the fluorescence parameters such as threshold or scale of the axis. Nonetheless, the instruments take up to three minutes for the reading and the analysis of one chips and thus can be potentially used in a clinical diagnostic setting.

## Additional file

Additional file 1: Figure S1. Review quality of dPCR chip. Representative images shown chips with good quality as data points above threshold or by calls (upper) and chips below the sufficient quality for miRNAs detection (lower). The data points in the plot are color-coded according to the following call types: FAM (blue), VIC (red), FAM + VIC (green) and NOT AMPLIFIED (yellow). (BMP 1250 kb)
