# Supplementary figures and images for: Novel method to detect microRNAs using chip-based QuantStudio 3D digital PCR
Source: BMC Genomics. 2015 Oct 23;16:849. doi: 10.1186/s12864-015-2097-9 (PMC4619272; doi:10.1186/s12864-015-2097-9)

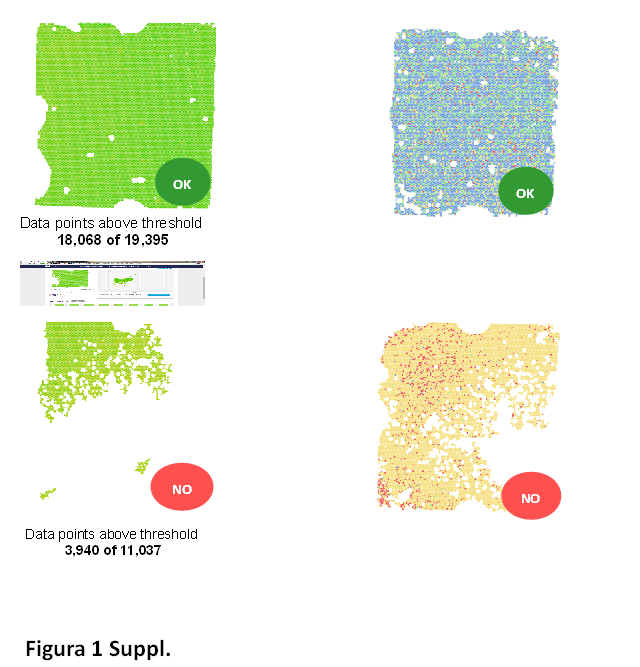

Supplement: Additional file 1: Figure S1. — Review quality of dPCR chip. Representative images shown chips with good quality as data points above threshold or by calls (upper) and chips below the sufficient quality for miRNAs detection (lower). The data points in the plot are color-coded according to the following call types: FAM (blue), VIC (red), FAM + VIC (green) and NOT AMPLIFIED (yellow). (BMP 1250 kb) [file 12864_2015_2097_MOESM1_ESM.bmp]
